# Analysis of Short-term Outcomes of Pancreatic Resections from a Low Volume Centre in a Tier II City in India

**DOI:** 10.1007/s13193-025-02325-5

**Published:** 2025-05-07

**Authors:** Amita Sekhar Padhy, Rajyalakshmi Puvvada, Rigved Nittala, Vishnu S. Menon, Sidaksingh R. Arora, Mounika Basani

**Affiliations:** Department of Surgical Oncology, Homi Bhabha Cancer Hospital & Research Centre, Visakhapatnam, 530053 Andhra Pradesh India

**Keywords:** Pancreaticoduodenectomy, Post-operative pancreatic fistula, Post-pancreatectomy haemorrhage, Biliary leaks, Low volume centre

## Abstract

Pancreatic cancer surgery represents the holy grail of hepatobiliary surgery and is the only option of curative treatment for malignancies involving this particular organ. This study aims to analyse the short-term outcomes of across the spectrum of surgeries performed for pancreatic neoplasms at a low volume hepatobiliary centre in eastern India. This is a retrospective study from our centre, from 1st January 2019 to 31st October 2024. Patients were identified from a prospectively maintained surgical database and electronic medical records, and data was collected from Electronic Medical Records. We identified 41 patients who underwent surgical resections during the study period. Median age was 56 years. Pre-operative biliary drainage was required in 24 (58.5%) cases. Pancreatico-duodenectomies represented with majority of cases (87.8%), followed by distal pancreas resections (2.4%), total pancreatectomy (2.4%) and ampullectomy (2.4%). Minimally invasive approach was attempted in 2 patients (4.9%). Post-operative complications and their incidences were post-operative pancreatic fistula (POPF) 26.8%, chyle leak 9.7%, biliary leaks 7.3%, delayed gastric emptying 19.4%, post pancreatectomy haemorrhage 4.8%, bowel-related complications 7.3, and surgical site infection 9.8%. Significant post-operative morbidity occurred in 24.4% of cases. Perioperative mortality rate was 7.3%. Although a low volume centre, our results are comparable to published literature for low volume centres, though worse than high volume centres. Safe outcomes are achievable at low volume centres with trained and dedicated surgeons, anaesthesiologists and proper patient selection.

## Introduction

Pancreatic cancer remains one of the most fatal malignancies and is a critical issue in the global burden of disease. The 5-year overall survival rate of pancreatic cancer is poor (10%), as majority of the cases are confirmed at an advanced stage [1]. Based on GLOBOCAN 2020 estimates, pancreatic cancer remains a large part of the global burden of disease and ranks as the 12th most common malignancy (2.6% of all cancers) and the 7th leading cause of cancer mortality (4.7% of all cancers) [2]. Treatment of pancreatic cancer varies according to stage and pathology often requiring a multimodality approach ranging from surgery, chemotherapy, and radiation therapy. Widely described as technically challenging and surgically demanding, it is important to emphasise that pancreatic surgeries is not just Whipple Procedures [3]. The pancreatic surgeries represent a spectrum ranging from ampullectomies to complex vascular resections and offers the only curative option for any pancreatic malignancy [3]. In a country like India with a lower incidence of pancreatic cancer than other parts of the world and marked disparity in quality care there has been a push to evolve regionalisation via dedicated training programmes [1, 4]. The reported perioperative mortality of about 5% in high volume centres though set as bench mark, it is important to note that associated with morbidity can reach up to 40% even at said high-volume centres [5]. Common complications following surgery are post-operative pancreatic fistula (POPF), delayed gastric emptying (DGE), haemorrhage, wound infection and biliary leak [6]. To tackle these peri-operative and post-operative challenges there is a need for close interdisciplinary collaboration and systems to ensure quality management. It is a challenge for a resource constraint setup like ours, with limited interventional radiology and endoscopist backup to evolve into self sustaining system. Centralization of such procedures will not only overburden the existing high volume centres but also increase the care-gap. This makes it all the more important for upcoming centres like ours to contribute more, not only to cushion load of high-volume centres but also to ensure greater accessibility of such complex procedures across different regions in India. This study aims to analyse short-term outcomes in surgeries performed for pancreatic neoplasms from a low volume centre in Tier II city in India.

## Materials and Methods

### Study Design, Population and Exclusion Criteria

This was a retrospective study of all pancreatic resections performed for peri-ampullary and pancreatic tumours in HBCH&RC in Visakhapatnam from January 2019 to October 2024. A total of 41 patients underwent surgery during this period. The inclusion criteria which were applied included (1) pancreatic neoplasm, (2) treated with curative intent, (3) registered and operated between 1 st January 2019 and 31 st October 2024 and (4) age more than 18 years. The following exclusion criteria were applied: (1) recurrent disease at presentation, (2) prior surgery at outside centre, (3) unresectable or metastatic disease at the time of exploration.

### Preoperative Workup, Surgical Technique and Post-operative Practices

Data was retrieved from electronic medical records, including information on demographic and clinical data—gender, age, American Society of Anaesthesiology (ASA) score, pre-operative biliary drainage, pre-operative carbohydrate antigen 19–9 (CA 19–9) levels, bilirubin, albumin, neo-adjuvant therapy, surgical procedure, blood loss, duration of surgery, length of hospital stay, operative morbidity and mortality occurring within 30 days after surgery, readmissions, histopathologic type and margin status.

In addition to stage, baseline performance status and comorbidities were taken into consideration before planning surgery. A dedicated pancreatic protocol CT was done in all cases within 4–6 weeks of surgery to identify the lesion, its relation to surrounding structures, arterial and venous anatomy, positron emission tomography (PET) scan was done in high-risk equivocal or indeterminate imaging findings, markedly elevated CA 19–9, large primary tumours, large regional lymph nodes, excessive weight loss and extreme pain. Primary surgical resection was offered to patients with resectable tumours who had a performance status and comorbidity profile that was appropriate for a major abdominal operation. Staging laparoscopy was performed for cases with lesions in the pancreatic body and tail, markedly elevated CA 19–9, large primary tumours, large regional lymph nodes, excessive weight loss and extreme pain; patients with borderline resectable pancreatic cancers (BRPC) received either neoadjuvant chemotherapy (NACT), followed by surgery. Endoscopic retrograde cholangiopancreatography (ERCP) and biliary stenting were performed in patients with cholangitis, bilirubin > 15, poor nutritional status (as a bridge to surgery), deranged coagulation profile and BRPC planned for neoadjuvant therapy (NAT). Peri-operative management was carried out according to our institutional standard Enhanced Recovery After Surgery (ERAS) protocol.

All surgeries were performed under general anaesthesia with epidural anaesthesia. For open procedures, the abdomen was opened through a bilateral subcostal incision or midline incision. For peri-ampullary tumours and tumours in the head of the pancreas and uncinate, pancreaticoduodenectomy, either pylorus preserving (PPPD) or classical Whipple’s was performed. Ampullectomy was performed for ampullary tumours amenable to local resection. For body and tail lesions, distal pancreatico-splenectomy (DPS) was performed. After PD, PJ (pancreatico-jejunostomy) was performed in a duct-to-mucosa, end-to-side fashion with four-layer interrupted sutures or PG (pancreatico-gastrostomy) via a dunking anastomosis. Naso-jejunal tube (NJT) is placed in the jejunum for early post-operative enteral feeding. Two drains are placed routinely, one posterior to hepaticojejunostomy (HJ) and PG/PJ, and the other drain is placed anterior to PG/PJ.

Post-operatively, patients were mobilized within 12 h, and urinary catheters were removed early. Chest physiotherapy and incentive spirometry were started immediately once the patient was fully awake and continued or escalated as appropriate until resolution of pulmonary issues and usual mobility, independence and exercise tolerance were restored. Adequate pain control in the post-operative period was achieved either by epidural analgesia, systemic opioids or analgesics provided by a dedicated pain team. On post-operative day 1, NJ feeds were started at 20 mL/h, and oral sips were allowed. On post-operative day 2, NJ feeds were increased to 40–60 mL/h, and clear liquids were allowed orally. On post-operative day 3, NJ feeds increased to 80 mL/h, and a liquid diet was allowed orally. From post-operative day 4, a soft diet was started, and NJ feeds were gradually weaned off. On post-operative days 3 and 7, serum and drain amylase levels were analysed to identify POPF, and drains were removed based on amylase levels, volume and colour of effluent.

### Defining Variables

We analysed the 30-day post-operative morbidity and mortality, retrospectively. Standard definitions were used for the classification of complications, especially pancreatic leaks given by the International Study Group of Pancreatic Fistula (ISGPF) [7], bile leaks by the International Study Group of Liver Surgery (ISGLS) [8], chyle leaks [9], post-pancreatectomy haemorrhage [10] and delayed gastric emptying (DGE) [11] by the International Study Group on Pancreatic Surgery (ISGPS).

### Statistical Analysis

Risk for POPF was calculated as per the Fistula Risk Score [12]. Statistical analysis was performed using SPSS version 27. Categorical variables were presented as numbers with percentages.

## Results

### Demographic Data

During the study period, total 41 patients underwent surgery for peri-ampullary and pancreatic tumours. The demographic data is summarised in Table 1.

**Table 1:** Demographic data (*n* = 41)

Mean age in years	52.24			
Sex	Male		18 (43.9%)	
	Female		23 (56.1%)	
American Society of Anaesthesiology grade	I		13 (31.7%)	
	II		25 (61.0%)	
	III		3 (7.3%)	
Mean body mass index in kg/m^2^	22.48			
Mean CA 19–9 levels in U/mL	30.00			
Mean pre-op bilirubin levels in mg/dL	2.52			
Mean pre-op haemoglobin levels in g/dL	11.2			
Mean pre-op albumin in g/dL	3.79			
Pre-operative biliary drainage (ERCP + Plastic stent)	Yes	24 (58.5%)		
	No	13 (41.5%)		
Neoadjuvant therapy (NAT)	Yes	4(9.8%)		
		NACT		3 (7.3%)
		NACTRT		1 (2.4%)
	No	37 (90.2%)		

*kg/m*^*2*^ kilogram per metre square, *CA 19.9* carbohydrate antigen 19–9, *mg/dL* milligram per decilitre, g/dL gram per decilitre, *ERCP* endoscopic retrograde cholangiopancreatography

NAT was given in 4 cases, 3 cases—NACT (FOLFIRINOX) and 1 case–NACTRT (received elsewhere)

### Surgical Details

Open approach was used for all the procedures except DPS which was performed laparoscopically (Table 2). Artery first approach is used in cases with doubtful resectability. There were no vascular resections. Total pancreatectomy was performed in one case in view of persistent positive margins on frozen. Pancreatic reconstruction was either PG/PJ based on surgeon preference. Hepaticojejunostomy was performed with intermittent sutures if CBD was not dilated and wall was thin.

**Table 2:** Surgery details (*n* = 41)

Surgical approach	Open	39 (95.1%)
	Lap	2 (4.9%)
Surgery type	PPPD	33 (80.5)
	Classical Whipple’s	3 (7.3%)
	DP with splenectomy	2 (4.9%)
	Spleen preserving DP	1 (2.4%)
	Total pancreatectomy	1 (2.4%)
	Ampullectomy	1 (2.4%)
Type of pancreatic anastomosis	PG	20 (48.8%)
	PJ	16 (39%)
	No anastomoses	5 (12.2%)
Type of bile duct anastomoses	Interrupted	31 (75.6%)
	Continuous	6 (14.6%)
	No Anastomoses	4 (9.8%)
CR FRS	Negligible	0 (0%)
	Low	7 (17.1%)
	Intermediate	28 (68.3%)
	High	6 (14.6%)
Simplified FRS	A	25 (61%)
	B	2 (4.9%)
	C	12 (29.3%
	D	2 (4.9%)
Median hospital stay in days	13 (8–62)	
Median ICU stay in days	2 (1–5)	
Mean blood loss in mL	852.07	
Mean blood replaced in mL	460.22	

PPPD pylorus preserving pancreatoduodectomy, Classical Whipple’s, *DPS* distal pancreatectomy, *PG* pancreatico-gastrostomy, *PJ* pancreaticojejunostomy, *FRS* fistula risk score, mL millilitre, *CR-FRS* clinically relevant fistula risk score

### Immediate Post-operative Complications and Management

All POPFs were managed conservatively with prolonged drainage or by radiologic intervention to drain collections (Table 3). In three patients who developed bile leak, one was managed with prolonged drainage; two underwent re-exploration and redo anastomosis (one out of the two expired). One patient, who developed post-pancreatectomy haemorrhage, required embolization of GDA pseudoaneurysm. This same patient later had GDA stump blowout requiring exploration and ligation. Chyle leaks were managed conservatively with a fat-free diet and prolonged drainage. Mortality occurred in two cases during same admission period and one after 1 st readmission. One case had haemorrhagic shock due to GDA stump blowout, one due to biliary peritonitis, and one had septic shock caused by necrotic bowel. There were two readmissions: one for post-pancreatectomy haemorrhage requiring exploration and one for surgical site infection, which was managed by dressings, antibiotics and re-suturing. Five patients developed sepsis with MODS requiring prolonged ventilatory support and upgradation of antibiotics. One patient had a GJ leak requiring re-exploration and redo anastomoses.

**Table 3:** Post-operative complications (*n* = 41)

Pancreatic fistula (26.8%)	Biochemical leak	2 (4.9%)	
	POPF B	9 (21.95%)	PJ 6 (14.63%)
			PG 3 (7.31%)
	POPF C	0 (0%)	
Biliary leak (7.3%)	Grade A	0 (0%)	
	Grade B	1 (2.4%)	
	Grade C	2 (4.8%)	
Chyle leak (9.7%)	Grade A	0 (0%)	
	Grade B	4 (9.7%)	
	Grade C	0 (0%)	
Delayed gastric emptying (19.4%)	Grade A	1 (2.4%)	
	Grade B	6 (14.6%)	
	Grade C	1 (2.4%)	
Surgical site infections (9.8%)	Superficial	4 (9.8%)	
Post-pancreatectomy haemorrhage (4.8%)	Grade A	0 (0%)	
	Grade B	0 (0%)	
	Grade C	1 (2.4%)	PJ 1 (2.4%)
			PG 0 (0%)
Bowel related (7.3%)	Enterocutaneous fistula	2 (4.9%)	
	Small bowel necrosis	1(2.4%)	
Ileus (4.9%)	2 (4.9%)		
Malena (4.9%)	2 (4.9%)		
Sepsis and MODS (14.5%)	6 (14.5%)		
GJ leak	1 (2.4%)		
Burst abdomen	8 (19.4%)		
Mortality	3(7.3%)		

*POPF* post-operative pancreatic fistula, *GJ* gastrojejunostomy

The incidence of major complications (i.e., Clavien Dindo grade III and above) was 31.6%, and 30-day mortality was 7.3% during the same admission period (Table 4).

**Table 4:** Classification of post-operative complications as per Clavien Dindo grading [13] (*n* = 41)

Grade	*N* = 41
0	12 (29.3%)
I	1 (2.4%)
II	18 (43.9%)
IIIA	3 (7.3%)
IIIB	2 (4.9%)
IVA	0
IVB	5 (12.19%)
V	3 (7.3%)

## Discussion

Pancreatic leaks were a major cause of morbidity in our study (Table 5). The incidence of POPF B/C (26.8%) in the present study is slightly higher to the current literature reporting incidences between 11.1 and 22.0% [14]. All cases except one of pancreatic leak in our study were managed conservatively with prolonged drainage and USG/CT-guided aspiration of collections. In mild pancreatic leaks, conservative management yields excellent outcomes [15, 16]. Today, the trend is more for conservative or interventional therapy for pancreatic fistulas or intra-abdominal collections with, e.g., persisting intraoperative drain, total parenteral nutrition (TPN), somatostatin therapy or CT-controlled drainage [17].

**Table 5:** Pathology details

Tumour location	Ampulla	18 (43.9%)
	Head of pancreas	13 (31.7%)
	Distal common bile duct	1 (2.4%)
	Body, tail of pancreas	3 (7.3%)
	Duodenum	2 (4.9%)
	No malignancy	4 (9.8%)
Histology	Adenocarcinoma	26 (63.4%)
	Neuroendocrine tumour	3 (7.3%)
	Solid pseudopapillary epithelial neoplasm	4 (9.8%)
	Schwannoma	1 (2.4%)
	Pancreatitis	4 (9.8%)
	Tubular adenoma	2 (4.9%)
	Intraductal tubule-papillary neoplasm	1 (2.4%)
pT	pT1	15 (36.6%)
	pT2	17 (41.5%)
	pT3	3 (7.3%)
	Complete response	1 (2.4%)
pN	pN0	13 (31.7%)
	pN1	11 (26.8%)
	pN2	7 (17.1%)
Median LN harvested	19 (1–46)	
Mean LN positive	2.41	
Margin	R0	36 (87.8%)
	R1	5 (12.2%)
	R2	0 (0%)

The incidence of chyle leak in our study is 9.7%, which is in line with other studies. All the cases were managed conservatively with a fat-free diet and prolonged drain. The incidences of post-pancreatectomy chyle leak varied from 1.3 to 22.1% in a comprehensive systematic review that included a total of 23 articles [18]. It is usually treated by a fat-free diet with no further adverse consequences. A recent meta-analysis comparing TPN with early enteral nutrition found the latter to be safe and substantially shorten the length of hospital stay of patients [19].

There were three (7.2%) patients with biliary leaks in our study. All except one patient were managed conservatively with prolonged drainage; two required re-exploration and redo anastomosis, and one of the two expired due to biliary peritonitis. In literature, the incidence of biliary leaks following pancreatic surgeries varied from 3.6 to 7.9% [20–22]. Early biliary leaks are usually a result of technical failure, either due to surgeon inexperience, separation between sutures, ischemia due to placement of the anastomosis distally on the hepatic duct, microvascular ischemia due to a close distance between sutures and tears in the duct due to traction from the sutures [23]. Late biliary leaks are usually secondary to POPF, systemic stress or localized fluid accumulations [24]. To avoid early leaks, a proper surgical technique with consideration of the texture and diameter of the hepatic duct seems paramount.

Post-pancreatectomy haemorrhage is rare, with a reported prevalence of 2–18% [25, 26]. The incidence of post-pancreatectomy haemorrhage in our study is 2.4%, which is consistent with what has been reported in the literature. This patient had hemodynamic instability requiring angiographic embolization of GDA pseudoaneurysm, later he developed GDA stump blowout for which re exploration and stump ligation was done. Another episode of massive haemorrhage was there after which this patient could not be salvaged. Some of the strategies to reduce post-pancreatic haemorrhage are pancreatic anastomosis with a small jejunal incision, falciform ligament wrap around the gastroduodenal artery stump and pancreatojejunostomy [27].

Our mortality rate is 7.3% (Table 6). All three patients underwent re-exploration for various complications ie, due to post-pancreatectomy haemorrhage, biliary leak (biliary peritonitis) and small bowel necrosis (sepsis). This is higher than those reported by large-volume centres but comparable to reports from other medium and low volume centres. As our experience increases, both with the surgery and critical care management along with better intervention radiology support, the mortality rates had improved in the past 2 years. However, it is too early to make such generalization and further measures like better case selection, standardisation of step and audit of outcomes are needed to improve the outcomes.

**Table 6:** Comparison of mortality rates with other studies

Author	Sample size, *N*	Mortality rate in percentage
Greenblatt et al. [28]	4945	2.6%
Narayanan et al. [29]	551	1.1%
Gungor et al. [30]	283	18%
Vinchurkar et al. [31]	26	3.84%
Jakhmola et al. [32]	69	11%
Jagannath et al. [33]	144	6.3%
Balachandran et al. [34]	218	9.6%
Our study	41	7.3%

### Hospital Volumes

To be able to complete PD, surgeons must accrue experience through repetition of the operative steps. Some have argued that PD outcomes are less dependent on hospital volume and more dependent on the technical competence of the operating surgeon [35]. Numerous authors have demonstrated the association between increasing surgeon volume and improved PD outcomes [36–38]. What is low volume and high volume is very fluid and different from study to study. A surgeon and team who perform complex operations several times a week will be more prepared to handle the complexities of a difficult case than a surgeon and team who perform PD several times a year. The determinants of outcome after PD are multifactorial and depend on hospital experience, surgeon experience and infrastructure availability.

Many PDs were safely performed in community hospitals by surgeons with varying degrees of experience with a comparably low mortality rate [43, 44], and many patients are dependent on such Tier II city referral hospitals.

Based on the above results, pancreatic resections can be performed in a low-volume centre without compromising patient safety and quality of care with comparable short-term results (Table 7). We believe pancreatic resections can be introduced to low-volume centres to ease the burden of surgery (from the High Volume Centres) for patients, safely in line with prior studies. Training of other personnel, such as anaesthetists, operating nurses and nurses in the wards for enhanced recovery after surgery (ERAS) fast track principles for improved perioperative outcomes, is essential.

**Table 7:** Summary of studies comparing perioperative mortality according to hospital volumes

Author	Peri-operative mortality		Study population	Definition of volume (surgeries per year)				
	Low volume	High volume		Very low	Low	Medium	High	Very-high
Balzano et al. [39]	8.1%	4.4%	Multicentre—7631 PDs	0–10	10–25	25–60	60–166	> 167
Krautz et al. [40]	10.4%	8.1%	National database—60858 PDs	< 8	8–18	19–31	32–58	> 59
Briceno et al. [41]	5.5%	2.6%	National database—19024 PDs	-	< 10	10–20	> 20	–
La Torre et al. [42]	2.5%	2.1%	Systematic review of 18 studies	-	< 9	9–12	13–18	> 19

We feel that the presence of following factors can help in delivering safe outcomes after pancreatic resections (Table 8).

**Table 8:** Factors for delivering safe outcomes after pancreatic resections

Surgeon trained in hepatobiliary and pancreatic and GI surgery
ERAS protocol
Anaesthesia and critical care team trained in GI surgery
Trained nursing staff

### ERAS Protocol for Gastrointestinal, Hepatobiliary and Pancreatic Surgeries

1. Starting Physiotherapy and Nutritional Rehabilitation atleast two weeks prior to surgery is an essential part that helps in early recovery from the surgical stress. DVT prophylaxis in the form of stocking and Low Molecular Weight Heparin 12h prior to surgery is a must. Early removal of tubes (Nasogastric, Foley, drains), Early enteral feeds help in early mobilisation of the patient.
2. **Experience at Homi Bhabha Cancer Hospital and Research Centre (HBCH&RC)**: Ours is a tertiary cancer centre under the Tata Memorial Centre (Mumbai), Department of Atomic Energy, Government of India. At our centre, proper patient selection, pre-operative assessment and treatment planning by a multidisciplinary team are the mainstays. We have a dedicated pre-habilitation and rehabilitation program that includes a team of physiotherapists, nutritionists and speech therapists. Managing challenges such as neutral fluid balance, avoidance of hypothermia, effective analgesia for post-operative respiratory function and post-operative complications requires team effort involving critical care specialists, surgeons trained in hepatobiliary and pancreatic surgery, ICU nurses and nutritional support services. We have achieved acceptable results at our centre due to this collaborative approach.

### Looking Forward

Future studies should investigate the long-term outcomes of pancreatic resections, including survival rates and quality of life from low-volume cancer centres. Additionally, there is a need for strategies to improve the surgical care for patients in such centres, such as training programs for surgeons, recruitment of specialists (interventional radiologists) and investment in surgical equipment.

Through the NCG (National Cancer Grid) and its member institutions, it is the right time to develop the cut-off for high-volume and low-volume centres for pancreatic resections corresponding to our disease burden, resources and constraints. Also, uniform maintenance and reporting of outcomes after pancreatic resections should be encouraged to create a national database under the NCG.

One of the main limitations of this study is the retrospective design, as well as the small sample size, and data regarding potential predictors of peri-operative outcomes such as fistula risk score was missing in some cases. Also, the study focused on short-term outcomes and complications, without assessing long-term factors like survival and quality of life.

## Conclusion

Pancreatic resections represent a spectrum of surgical procedures and are associated with a wide-range of complications which are challenging in low volume centres. Although a low volume centre, our results are comparable to published literature for low volume centres. However, with experience gained, standardization of procedure and inter-disciplinary collaborations we can expect improved short-term outcomes in the coming years.

## Data Availability

The data was retrieved from Project Number 12000095, after due approval from the Institutional Ethics Committee. Upon due request, further details can be provided by the authors.
